# Prognostic factors for pain chronicity in low back pain: a systematic review

**DOI:** 10.1097/PR9.0000000000000919

**Published:** 2021-04-01

**Authors:** Linda Karoliina Nieminen, Liisa Maria Pyysalo, Markku Juhani Kankaanpää

**Affiliations:** a Department of Rehabilitation and Psychosocial Support, Tampere University Hospital, Tampere, Finland; b University of Tampere, Tampere, Finland

**Keywords:** Nonspecific, Low back pain, Risk factors, Prognostic factors, Chronic pain

## Abstract

Low back pain is the leading cause for years lived in disability. Most people with acute low back pain improve rapidly, but 4% to 25% of patients become chronic. Since the previous systematic reviews on the subject, a large number of new studies have been conducted. The objective of this article was to review the evidence of the prognostic factors behind nonspecific chronic low back pain. A systematic literature search was performed without date limitation from the MEDLINE, Cochrane library, and Medic databases. Specific inclusion criteria were used, and risk factors before the onset of chronic symptoms were searched. Study quality was assessed by 2 independent reviewers. One hundred eleven full articles were read for potential inclusion, and 25 articles met all the inclusion criteria. One study was rated as good quality, 19 studies were rated as fair quality, and 5 articles were rated as poor quality. Higher pain intensity, higher body weight, carrying heavy loads at work, difficult working positions, and depression were the most frequently observed risk factors for chronic low back pain. Maladaptive behavior strategies, general anxiety, functional limitation during the episode, smoking, and particularly physical work were also explicitly predictive of chronicity. According to this systematic review, several prognostic factors from the biomechanical, psychological and psychosocial point of view are significant for chronicity in low back pain.

## Abstract 2

Supplemental Digital Content is Available in the Text.

Several prognostic factors are related to low back pain chronicity, and these should be taken into account when planning more comprehensive models in its prevention.

## 1. Introduction

Low back pain (LBP) is the leading cause of years lived in disability in high-income and middle-income countries.^39^ Moreover, a similar increase has also been seen in low-income countries.^68^ In 2015, LBP was responsible for approximately 60.1 million years lived in disabilities, an increase of 54% since 1990.^39^ For industrialized countries, LBP is a very costly illness^21,138^ and indirect costs (work absenteeism, productivity loss) account for more than half of the total costs.^9^ In many patients, the specific nociceptive source of LBP cannot be identified and those affected are often classified as having so-called “nonspecific low back pain.”^84^ Nonspecific LBP represents 90% to 95% of cases, with other causes being specific spinal pathology (<1% of cases) and radicular syndrome (approximately 5%–10% of cases).^7^ The global point prevalence of activity‐limiting LBP lasting more than 1 day is estimated to be 12%.^69^ Although most patients with acute LBP show rapid improvements in pain and disability within 1 month,^106^ between 4% and 25% of patients drift to chronicity.^92^ The prevalence of chronic low back pain (CLBP) increases linearly from the third decade of life until the age of 60 years, with CLBP being more prevalent in women.^92^

The prognosis of nonspecific LBP is greatly influenced by factors not related to the spine.^115^ In 1987, a biopsychosocial model for understanding LBP was first introduced by George Waddell.^136^ The idea behind the model is based on how psychologic and social influences modulate an individual's perception of symptoms. An overemphasis on pain alone and a dependence on only mechanical, nominal diagnosis can lead to more disability. Therefore, when treating patients with LBP, clinicians should consider all aspects (biomechanical, psychological, and psychosocial) of the illness.

To date, few comprehensive reviews have studied the risks of chronicity in patients with LBP. A review by Valat et al. in 1997^133^ concluded that CLBP is more closely related to demographic, psychosocial, and occupational factors than to the medical characteristics of the disorder itself. A 2010 systematic review of “yellow flag” risk factors for developing CLBP^15^ concluded that maladaptive pain coping behaviors, lower functional impairment at baseline, nonorganic signs referring to somatization, worse general health status before the onset of pain, and the presence of psychiatric comorbidities were significant in terms of chronicity. Since then, a large number of studies have focused on revealing the risk factors behind this global problem.

The aim of this systematic review is to identify the prognostic factors for pain chronicity in patients with LBP and to provide an update on the existing data.

## 2. Materials and methods

### 2.1. Literature search

Systematic literature searches from computerized databases were conducted until March 30, 2020. The search strategy was developed in collaboration with an information specialist. The following databases were searched without any date restriction: MEDLINE (PubMed), Cochrane Database, and Medic specifically for articles in the Finnish language. The primary target of the search was articles concerning predictive risk factors for chronic, nonspecific LBP. The full search strategy is presented in Appendix 1 (available at http://links.lww.com/PR9/A99).

### 2.2. Study selection and inclusion criteria for selection of studies

The study types included in the literature search were cohort studies, follow-up studies, and reviews. The reviews were used only to search for additional articles to avoid duplication. Randomized controlled trials were not included because the effect of the intervention on the outcome (CLBP) could not be excluded and observing only the group without intervention could create bias. However, studies with interventions could be included if the intervention concerned the whole followed population or its impact could be taken into account in some other way. The references of the studies that met the inclusion criteria were searched for additional articles. There was no time limit for the search. Studies in the English or Finnish languages that focused on working population (aged 18–65 years) were included. If older individuals were recruited, the mean age with SD had to be no more than 65 years. The main outcome was nonspecific CLBP with or without pain radiation, but specific nerve root disorders were excluded. Articles that dealt only with operative treatment were also excluded. Chronic pain is most commonly described as lasting longer than 3 months.^129^ Therefore, studies must have assessed the predictive risk factors before that period to be included in the search. A chronic condition was defined as persistent pain in the lower back for a period of 3 months or longer.

### 2.3. Quality assessment

Study quality was assessed using the National Institute of Health study assessment tool.^94^ Two independent reviewers evaluated all the included articles according to assessment tool criteria. If the ratings differed, the reviewers discussed the article in an effort to reach consensus. If consensus was not achieved, a third reviewer was consulted. Each study was judged as good, fair, or poor by evaluating the potential risk of bias resulting from the existing flaws.

## 3. Results

### 3.1. Results of the search

**Figure 1.**
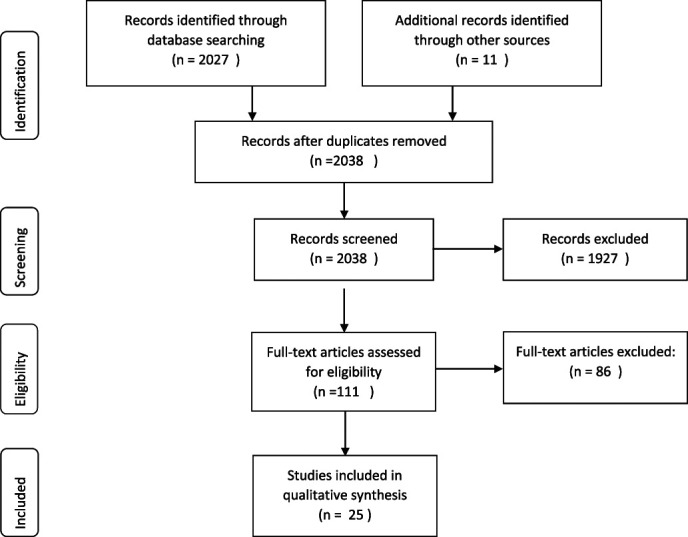
Prisma flow diagram^93^ of the study selection process.

A Prisma flow chart of the study selection is presented in Figure 1. A total of 2,028 articles were identified. The first exclusion round was based on inappropriate titles or abstracts. We then read the full text of 111 articles, and 25 articles met all the inclusion criteria. Characteristics of the included studies are presented in Table 1. Of these 25 articles, 17 68% were published in 2010 or thereafter.^32,56–63,83,88,89,97,99,103,119,122^ Two articles were found from the references of included articles.^46,55^ The excluded articles and the reasons for exclusion are listed in Table 2. Most of the excluded articles did not meet the criteria concerning the prospective information before the onset of chronic pain, the chronic pain was defined as lasting less than 3 months/12 weeks, or the pain was already chronic at baseline. In some articles concerning the working population, the chronic disease was only defined according to the time spent on sick leave without explaining whether the sick leave was due to LBP or to some other medical condition. In many of the excluded articles, the outcome was defined as timely pain during the follow-up contact compared with persistent symptoms for at least 3 months.

**Table 1:** Characteristics of included studies.

Author, year of publication, country	Study design	Study objective	Follow-up time	Population at follow-up	Inclusion and exclusion criteria	Participant characteristics	Chronic LBP after follow-up	Prognostic risk factors with significant *P*
Bakker et al. 2007,^6^ the Netherlands	Prospective inception cohort study	To assess the prognostic value of spinal mechanical load and influence on the course of acute LBP	6 mo	n = 88	Nonspecific LBP less than 6 wk, exclusion: pathologic and sciatica syndrome, not understanding Dutch language, previous episode of LBP in the past 12 mo, significant trauma, pregnancy, and spinal surgery	Age 15–82 y (mean 41, SD 13.5), 56% male, and mean duration of symptoms 11.8 d	n = 53 (60%)	Smoking OR 4.41 95% CI 1.50–12.95, age OR 0.96 95% CI 0.93–0.99
Coste et al. 2003,^18^ France	Inception cohort study	To investigate various biologic and psychosocial factors in the natural history of acute LBP	3 mo	n = 111	18 y or older, primary complaint of LBP, and pain duration <72 h without radiation below the gluteal fold. Exclusion: malignancy, infection, spondyloarthropathy, vertebral fracture, neurologic signs, or episode of LBP during the previous 3 mo, illiteracy, or unable to speak French	Age ≥18 y (mean 44.3, SD 13.7), 49% male, and mean duration of symptoms 1.1 d	n = 6 (5%)	Poorer disability at baseline recovery HR 0.97 95% CI 0.93–1.00 (*P* = 0.05) and poorer general health at baseline recovery HR 0.89 95% CI 0.80–0.99 (*P* = 0.03)
Coste et al. 1994,^19^ France	Inception cohort study	To identify clinical, psychological, and sociodemographic prognostic factors for recovery from acute LBP	3 mo	n = 92	18 y and over, primary complaint back pain, and duration <72 h without radiation below gluteal fold. Exclusion: malignancies, infections, spondyloarthropathies, vertebral fractures, neurological signs, or episode of LBP during the previous 3 mo, illiteracy, or unable to speak French	Age ≥18 y (mean 46.5, SD 14.3), 60% male, and mean duration of symptoms 26 h	n = 2 (1.9%)	Previous chronic episode of LBP HR for recovery 0.21 95% CI 0.07–0.60 (*P* = 0.0004) and pain worse on standing 0.49 95% CI 0.30–0.77 (*P* = 0.003)
Esquirol et al. 2016,^32^ France	Prospective cohort study (VISAT study)	To determine the impact of a wide range of occupational factors on the incidence and persistence of chronic LBP	5 y	n = 1560	Workers born in 1934, 1944, 1954, and 1964	Age 32–52 y, 52% male	n = 255 (22.6%)	Older age 42 y OR 1.44 95% CI 1.02–2.03 and 52 y 1.46 95% CI 0.99–2.15, history of rheumatological events ≥1 OR 2.34 95% CI 1.69–3.25, former productivity-related income 2.03 95% CI 1.18–3.50, number of different jobs held ≥2 OR 0.70 95% CI 0.51–0.95, carrying heavy loads at work OR 1.54 95% CI 1.09–2.18, and nonrecognition of work OR 1.76 95% CI 1.21–2.56
Hagen et al. 2005,^46^ Norway	Public health study (HUNT studies)	To evaluate the relationship between blood pressure and prevalence of chronic MSCs	11 y	n = 46901	All residents of the county 20 y and older	Age ≥20 y	n = 8182 (17.5%)	Higher blood pressure OR 0.7 95%CI 0.6–0.7
Heneewer et al. 2007,^54^ Belgium	Prospective cohort study	To evaluate the association between psychosocial factors and the transition from acute to subacute LBP to chronicity	3 mo	n = 56	New episode of nonspecific LBP less than 12 wk, pain-free period at least 3 mo, age between 21–60 years, and able to understand the Dutch language. Exclusion: suspicion of specific cause, pregnancy, and coexisting major medical disease.	Age (mean) 41.95 y, 61% male, and duration of symptoms <4 wk 52%, 4–6 wk 27%, 7–12 wk 21%	n = 25 (45%)	Higher pain intensity OR 1.787 95% CI 1.677–1.916 (*P* = 0.002)
Henschke et al. 2008,^55^ Australia	Cohort study	To estimate 1-y prognosis and identify prognostic factors in cases of recent-onset LBP managed in primary care	1 y	n = 944	Low back pain 24 hours—2 wk, at least 14 years old, able to speak and read English. Exclusion: serious pathology, radiculopathy	Age (mean) 43.3 y (SD 14.4), 54.8% male, and mean duration of symptoms 4.9 d	n = 388 (41%)	Age recovery HR 0.99 95% CI 0.99–1.00 (*P* = 0.004), pain intensity recovery HR 0.86 95% CI 0.77–0.96 (*P* = 0.009), depression recovery HR 0.94 95% CI 0.91–0.97 (*P* < 0.001), risk of persistence recovery HR 0.92 95% CI 0.89–0.95 (*P* < 0.001), compensable LBP recovery HR 0.59 95 %CI 0.47–0.74 (*P* < 0.001), days of reduced activity recovery HR 1.04 95% CI 1.00–1.008 (*P* = 0.033), and duration of episode recovery HR 0.97 95% CI 0.94–1.0 (*P* = 0.033)
Herin et al. 2014,^56^ France	Longitudinal prospective epidemiological survey (ESTEV)	To assess the impact of work-related factors according to sex on the development of regional and multisite MSP	5 y	n = 12591	Workers born in 1938, 1943, 1948, and 1953, random selection from patients under the supervision of volunteer physicians	Birth year 1938 16.9%, 1943 27%, 1948 28.4%, 1953 27.7%, male 64.8%, BMI ≥25 43.4%, blue collar workers 25.4%, clerks 26.5%	n = 1206 (9.6%)	Forceful effort at work HR 1.20 95% CI 1.01–1.44 men, awkward postures HR 1.19 95% CI 1.01–1.39 men, HR 1.33 95% CI 1.07–1.64 women, and exposure to vibration HR 1.73 95% CI 1.01–3.01 women
Heuch et al. 2019,^57^ Norway	Follow-up study (HUNT studies)	To explore the association between diabetes and subsequent risk of chronic LBP	11 y	n = 18972	All residents of the county 20 y and older, study was restricted to respondents aged 30–69 y, and without chronic LBP at baseline and with known information about diabetes	Age 30–69 y	n = 3380 (17.8%)	Diabetes men RR 1.43 CI 95% 1.04–1.96 (*P* = 0.043)
Heuch et al. 2017,^58^ Norway	Prospective cohort study (HUNT studies)	To study association between physical activity level at work and risk of chronic LBP	11 y	n = 14915	All residents of the county 20 y and older, study was restricted to respondents aged 30–69 y. Study included participants without chronic LBP at baseline, with information about physical activity at work, education, physical activity in leisure time, smoking, and BMI. Exclusion: not employed or did not perform professional work	Age 30–69 y, 49% male	n = 2501 (16.8%)	Particularly strenuous physical work men RR 1.22 95% CI 1.01–1.49 (*P* = 0.041) and work involving walking and heavy lifting women RR 1.21 95% CI 1.06–1.38 (*P* = 0.006))
Heuch et al. 2015a,^59^ Norway	Cohort study (HUNT studies)	To compare relationships with LBP for several measures of body size	11 y	n = 25329	All residents of the county 20 y and older, study was restricted to respondents aged 30–69 y, with information whether they suffered from chronic LBP and had measurements of height, weight, waist, and hip	Age 30–69 y, 50% male, and 74% without LBP at baseline	NA	Body weight (kg):RR 1.087 95% CI 1.039–1.138 women (*P* < 0.001), RR 1.091 95% CI 1.030–1.157 men (*P* = 0.003), BMI: RR 1.075 95% CI 1.023–1.128 women (*P* = 0.004), RR 1.091 95% CI 1.027–1.158 men (*P* = 0.004), higher hip and waist circumference; waist RR 1.078 95% CI 1.025–1.134 women (*P* = 0.004), 1.064 95% CI 1.001–1.131 men (*P* = 0.05), hip: RR 1.073 95% CI 1.024–1.123 women (*P* = 0.003), 1.060 95% CI 1.00–1.123 men (*P* = 0.05)
Heuch et al. 2015b,^60^ Norway	Prospective cohort study (HUNT studies)	To study associations between body height and chronic LBP	11 y	n = 25329	Cohort of population aged 30–69 y with or without LBP	Age 30–69 y, 45% male, and 74% without LBP at baseline	n = 3230 (17%) of those without chronic LBP at baseline	Women height per 10 cm RR 1.09 95% CI 1.01–1.17 (*P* = 0.03)
Heuch et al. 2014a,^61^ Norway	Prospective cohort study (HUNT studies)	To study relation between levels of cholesterol, HDL, and triglycerides to chronic LBP	11 y	n = 25450	Cohort of population aged 30–69 y with or without LBP	Age 30–69 y, 45% male, and 74% without LBP at baseline	n = 3254 (17%) of those without chronic LBP at baseline	All results not significant statistically after complete adjustment for confounding variables
Heuch et al. 2014b,^62^ Norway	Prospective study (HUNT studies)	To investigate associations between blood pressure and chronic LBP	11 y	n = 22949	Cohort of population aged 30–69 y with or without LBP	Age 30–69 y,45% male, and 75% without LBP at baseline	n = 2936 (17%) of those without chronic LBP at baseline	Higher systolic pressure OR 0.95 95% CI 0.92–0.99 women (*P* = 0.005) and pulse pressure OR 0.93 95% CI 0.89–0.98 women (*P* = 0.007)
Heuch et al. 2013,^63^ Norway	Prospective cohort study (HUNT studies)	To determine whether elevated BMI increase chronic LBP	11 y	n = 25450	Cohort of population aged 30–69 y with information available on height, weight, and with or without chronic LBP at baseline	Age 30–69 y, 45% male, and 74% without LBP at baseline	n = 3254 (17%) of those without chronic LBP at baseline	BMI ≥30 vs BMI ≤25 OR 1.34 95% CI 1.08–1.67 men (*P* = 0.006), OR 1.22 95% CI 1.03–1.46 women (*P* = 0.008)
Machado et al. 2016,^83^ Australia	Case crossover study	To investigate the association of transient exposures to physical and psychosocial activities with the development of nonpersistent and persistent LBP	12 mo	n = 832	Sudden-onset LBP with or without leg pain, preceded by a period of at least 1 mo without LBP. Must comprehend English, presented within 7 d from pain onset, and pain at least moderate intensity. Exclusion: serious spinal pathology	Mean age 45.3 y, 54% male	n = 352 (42.3%)	Moderate or vigorous physical activity OR 2.4 95% CI 1.2–4.8, vigorous only OR 2.8 95% CI 1.0–7.8, manual tasks involving heavy loads OR 8.0 95% CI 2.8–22.6, awkward postures OR 16.0 95% CI 5.0–51.4
Mehling et al. 2015,^88^ USA	Prospective cohort study	To investigate the prognosis of acute LBP	2 y	n = 436	Age 18–70, pain less than 1 mo, no other episodes preceded in the past year, speaking English, no red flags, fibromyalgia, chronic pain conditions, disabling psychiatric disease, or prescription for narcotics	Average age 50.5(±12.6) years, 44% male, 61% with a college degree, 59% employed full time, and median duration of pain at baseline 14 d	n = 66 (13%) at 6 months, n = 84 (19%) at 2 y	At 6 mo: perceived risk that pain will persist OR 1.13 95% CI 1.01–1.27, catastrophizing OR 1.12 95% CI 1.01–1.24, coping with pain by ignoring OR 1.11 95% CI 1.01–1.21, coping with TV or music OR 0.90 95% CI 0.82–0.98, pain spreading to the upper back OR 6.06 95% CI 2.98–12.31; at 2 y: perceived stress OR 1.12 95% CI 1.02–1.24, low willingness to tolerate pain OR 1.17 95% CI 1.00–1.36
Melloh et al. 2013,^89^ Australia	Inception cohort study	To evaluate risk factors and protective factors of persistent LBP	6 mo	n = 168	Cohort consecutively recruited by health practitioners. Ability to read and write English, 18–65 y. Exclusion: LBP free at baseline, chronic LBP at baseline, specific LBP, osteoarthritis of knee or hip, pregnancy, and age older than 65 y	Mean age 36.0 y (±13.1), 48% male, mean BMI 28 (±6)	n = 38 (23%)	Social support at work OR 0.67 95% CI 0.45–0.99 (*P* = 0.045), somatization OR 1.08 95% CI 1.01–1.15 (*P* = 0.022)
Nilsen et al. 2011,^97^ Norway	Prospective study of longitudinal data (HUNT studies)	To investigate the association between physical exercise, BMI, and risk of chronic MSP	11 y	n = 32417	All residents of the county 20 y or older, patients who participated at baseline and follow-up, had all relevant baseline information available. Exclusion: MSP for 10 y or more, physically impaired at baseline	48% male, mean BMI 24.9 (±27.7)	n = 3314 (10.2%)	Physical exercise ≥2 h/wk RR 0.92 95% CI 0.79–1.07 women (*P* = 0.02), RR 0.75 95% CI 0.64–0.88 men (*P* < 0.001), and obesity RR1.21 95% CI 1.04–1.41 women (*P* < 0.001)
Nolen et al. 2017,^99^ Canada	Population-based cohort study	To investigate the association between a lifetime history of LBP injury in a motor vehicle collision and future troublesome LBP	12 mo	n = 509	Saskatchewan residents 20–69 years old with a valid health services card. Age-stratified random sample of 0%. 4% from eligible individuals	Mean age 40,4 y (SD 12.5), 58% male, and history of low back injury 6.1%	n = 45 (at 6 mo, 7.6%) and n = 39 (at 12 mo 7.7%)	History of low back injury in a motor vehicle collision HRR = 2.20, 95%CI 1.04–4.68
van Oostrom et al. 2012,^103^ the Netherlands	Prospective cohort study	To explore long-term associations between physical load exposure and chronic LBP	10 y	n = 4378	Inhabitants of Doetinchem, 20–60 y, were examined in population-based study every 5 y for 15 y, this study used population from the second examination onward	Age 25–65 y, 46.6% male, at paid job 61.8%, smokers 31.1%, and BMI ≤25 49.3%	n = 3196–3230 (20%)	Awkward postures OR 2.51 95% CI 1.25–5.07
Poiraudeau et al. 2006,^110^ France	Longitudinal descriptive survey	To assess the outcome of subacute LBP, identify characteristics related to outcome of patients and physicians	3 mo	n = 440 (patients). n = 266 (physicians)	Random selection of rheumatologists from national database, each enrolled 1–4 consecutive patients. Exclusion: ≤18 y, had pain less than 4 or more than 12 wk, sciatica, subacute LBP during the past 12 mo, unemployed, pregnancy, infection, tumor, of inflammatory disease, and had consulted another physician for the same episode	Patients: mean age 42.8 y (±9.5), 58.4% male, and duration of back pain 6.1 wk (±1.6)	n = 178 (40%)	Anxiety OR 2.41 95% CI 1.44–4.09 (<0.001), female sex OR 2.03 95% CI 1.30–3.18 (*P* = 0.0033), work-related back pain OR 3.37 95% CI 1.08–5.17 (*P* = 0.0028), patients' beliefs about work-related back pain OR 1.02 95% CI 1.00–1.05 (<0.001)
Shaw et al. 2010,^119^ USA	Prospective cohort study	To assess whether pre-existing psychiatric diagnoses increase the likelihood of transitioning from subacute to chronic LBP	12 mo	n = 122	First episode of LBP lasting 6–10 wk, age 18–50 y. Exclusion: major medical illness, pain disorder, taking medications to affect mood, major surgery 12 mo earlier, back pain from neoplastic disease, and osteomyelitis or fracture	Average age 30 y (±7.19),59% psychiatric disorder, 46% back pain without radiation, 16% had neurological signs (weakness, reflex, or sensory abnormality)	n = 49 (40%)	Depression OR 4.99 95% CI 1.49–16.76 (*P* < 0.01), general anxiety OR 2.45 95% CI 1.06–5.68 (*P* < 0.05), post-traumatic stress disorder OR 3.23 95% CI 1.11–9.44 (*P* < 0.05), nicotine dependence OR 2.49 95% CI 1.15–5.40 (*P* < 0.05), and psychiatric comorbidity 3.21 95% CI 1.29–7.99 (*P* < 0.05)
Sihawong et al. 2016,^122^ Thailand	Prospective study	To identify predictors for chronic neck and LBP	1 y	n = 615	18–55 y working full time. Exclusion: Symptoms 3 mo before baseline, pregnancy, history of trauma in the spinal region, surgery 12 mo before baseline, and had diagnosis for specific disease of the spine	Mean age 35.7 (±8.3), 25% male, history of LBP 78.5%, and BMI 23.4 (±4.9)	n = 28 (26.7%)	History of LBP OR 4.54 95% CI 1.02–20.21 (*P* = 0.04), high initial pain intensity OR 1.82 95% CI 1.46–2.28 (*P* < 0.01)
Wand et al. 2009,^140^ United Kingdom	Prospective observational study	To evaluate which patient profile offers the most useful guide to long-term outcome in acute LBP	6 mo	n = 54	Nonspecific LBP less than 6 wk, 20–55 y, pain free at least 3 mo. Exclusion: specific low back pathology, nerve root pain, pregnancy or less than 3-mo postpartum, involvement in litigation, coexisting major medical disease, currently in physiotherapy, and previous spinal surgery	Mean age 35 y, range 21%–55%, 48% male, duration 2.9(±1.4) wk, and 93% employed	NA	LBP-related disability, RMDQ correlation coefficient 0.48 (*P* < 0.01), higher pain intensity correlation coefficient 0.40 (*P* < 0.01), quality of life, EQ5D correlation coefficient −0.42 (*P* < 0.01), physical well-being, PCS correlation coefficient −0.36 (*P* < 0.01)

BMI, body mass index, EQ5D, Euro-Qol health transition score, ESTEV study, French epidemiological survey, Health, Work, and Ageing investigation, HUNT study, Nord-Trondelag Health Study, LBP, low back pain, MSC, musculoskeletal complaint, MSP, musculoskeletal pain, PCS, Short Form-36 physical component score, RMDQ, Roland– Morris Disability Questionnaire, VISAT study, Viellissement Santé Travail study

**Table 2:** Excluded articles with reasons for exclusion.

Article	Reason for exclusion
Amorim et al.^3^	Only chronic population at baseline
Andersen et al.^5^	Baseline information inadequate
Andersen et al.^4^	Different definition for chronic pain; >30 days during last year
Ashworth et al.^2^	Including chronic population at baseline
Beneciuk et al.^8^	Including chronic population at baseline
Bohman et al.^10^	Different definition for chronic pain; no persistent pain
Burton et al.^11^	Including chronic population at baseline
Campbell et al.^12^	Including chronic population at baseline
Carey et al.^13^	Different definition for chronic pain; RMDQ
Cats-Baril and Frymoyer^14^	Baseline information inadequate
Chou and Shekelle^15^	Review
Costa et al.^17^	Only chronic population at baseline
Currie and Wang^20^	Different definition for chronic pain; no time frame, including adolescents
Dario et al.^22^	Baseline information inadequate
Diamond and Borenstein^23^	Dissertation
Dunn et al.^26^	Including chronic population at baseline
Edmond et al.^27^	Different definition for chronic pain; maximal pain over the past week
El-Metwally et al.^29^	Only chronic population at baseline
Endo et al.^30^	Baseline information inadequate
Esteve et al.^31^	Multiple pain sites
Fishbain et al.^33^	Only chronic population at baseline, multiple pain sites
Fransen et al.^35^	Baseline information inadequate
Friedman et al.^36^	Different outcome; Roland Morris disability questionnaire
Gatchel et al.^37^	Different definition for chronic pain; return to work status at follow-up
Gatchel et al.^38^	Different definition for chronic pain; return to work status at follow-up
Green et al.^40^	Including chronic at baseline
Grotle et al.^43^	Different definition for chronic pain; pain during the past week at follow-up
Grotle et al.^42^	Different definition for chronic pain; RMDQ at 12 mo
Gurcay et al.^44^	Different definition for recovery; assessed after 2 wk of follow-up
Hagen et al.^45^	Baseline information inadequate
Haglund et al.^47^	Only chronic population at baseline
Hasue and Fujiwara^48^	Baseline information inadequate
Hayden et al.^49^	Including chronic population at baseline
Hayden et al.^50^	Review (the part discussing population)
Heitz et al.^51^	Review
Helmhout et al.^52^	Including chronic population at baseline
Heneewer et al.^53^	Only chronic population at baseline
Heymans et al.^64^	Including chronic population at baseline
Holtermann et al.^66^	Different definition for chronic pain; >30 d during last year
Hussain et al.^70^	Baseline information inadequate
Imagama et al.^71^	Study on elderly
Jegan et al.^72^	Only chronic population at baseline
Jones et al.^73^	Including chronic population at baseline
Kardouni et al.^74^	Baseline information inadequate
Klenerman et al.^77^	Different definition for outcome; information on the chronic group inadequate
Kopec et al.^78^	Different definition for chronic pain; diagnose for back problems
Kovacs et al.^79^	Including chronic population at baseline
Lagersted-Olsen et al.^80^	Baseline information inadequate
Matsuda et al.^85^	Only chronic population at baseline
Matsudaira et al.^87^	Baseline information inadequate
Matsudaira et al.^86^	Baseline information inadequate
Melloh et al.^90^	Different definition for chronic pain; >6 wk, measured by oswestry
Mercado et al.^91^	Baseline information inadequate, multiple pain sites
Neubauer et al.^95^	Including chronic population at baseline
Nisenzon et al.^98^	Baseline information inadequate
Noormohammadpour et al.^100^	Only chronic population at baseline
Nordstoga et al.^101^	Only chronic population at baseline
Oliveira et al.^102^	Only chronic population at baseline
Pagé et al.^104^	Only chronic population at baseline
Picavet et al.^107^	Baseline information inadequate
Pinheiro et al.^108^	Only chronic at baseline
Pinto et al.^109^	Only chronic population at baseline
Popescu and Lee^111^	Dissertation
Rabey et al.^112^	Only chronic population at baseline
Ramond et al.^113^	Review
Reis et al.^114^	Baseline information inadequate
Rodeghero et al.^116^	Baseline information inadequate
Schiøttz-Christensen et al.^117^	Different definition for chronic pain: sickleave and functional recovery
Shiri et al.^121^	Review and meta-analysis
Shultz et al.^118^	Baseline information inadequate
Smedley et al.^123^	Baseline information inadequate
Swinkels-Meewisse et al.^126^	Different definition for chronic pain; point prevalence at follow-up
Thomas et al.^127^	Baseline information inadequate
Traeger et al.^128^	Duplicate
Trinderup et al.^130^	Only chronic population at baseline
Urquhart et al.^131^	Prevalence study, does not have a follow-up
Wahlgren et al.^137^	Different definition for chronic pain; point prevalence at follow-up
Valat et al.^132^	Different definition for chronic pain; 7 wk
Walton et al.^139^	Multiple pain sites
van der Hoogen^67^	Including chronic population at baseline
van der Weide et al.^141^	Different definition for chronic pain; functional disability, return to work
Verkerk et al.^134^	Only chronic population at baseline
Werneke et al.^142^	Different definition for chronic pain; pain during the past week at follow-up
Wilkens et al.^143^	Only chronic population at baseline
Villafañe et al.^135^	Only chronic population at baseline
Williams et al.^144^	Different definition for chronic pain; point prevalence at follow-up
Yosef et al.^146^	Including chronic population at baseline

### 3.2. Quality assessment

The methodological quality of the studies was evaluated. Only 1 study was rated as good quality,^46^ 19 studies were rated as fair quality,^6,18,32,54–63,89,97,99,119,122,140^ and 5 articles were rated as poor quality.^19,83,88,103,110^ Those studies that met the criteria according to the National Institute of Health assessment tool^94^ are categorized as study population, measured exposures, measured outcomes, and study characteristics in Table 3.

**Table 3:** Criteria for methodological quality.

Criteria for methodological quality	All articles n = 25 [n (%)]	Good n = 1 [n (%)]	Fair n = 19 [n (%)]	Poor n = 5 [n (%)]
Study population				
Description of population	20 (91)	1 (100)	17 (89)	4 (80)
Participation of eligible participants ≥50%	18 (82)	1 (100)	16 (84)	3 (60)
Inclusion criteria precise	21 (96)	1 (100)	19 (100)	4 (80)
Loss to follow-up ≤20%	7 (32)	0 (0)	7 (37)	1 (20)
Measured exposures				
Exposures measured before outcome	22 (100)	1 (100)	19 (100)	5 (100)
Levels of exposure examined	13 (59)	1 (100)	12 (63)	3 (60)
Exposure measures valid	10 (45)	1 (100)	9 (47)	0 (0)
Exposures assessed more than once	10 (45)	1 (100)	8 (42)	1 (20)
Measured outcome				
Sufficient timeframe to detect outcome	22 (100)	1 (100)	19 (100)	5 (100)
Outcome measures valid	8 (36)	1 (100)	7 (37)	1 (20)
Study characteristics				
Research question clearly stated	19 (86)	1 (100)	18 (95)	3 (60)
Sample size justification	3 (14)	1 (100)	2 (11)	0 (0)
Outcome assessors blinded	1 (5)	0 (0)	1 (5)	0 (0)
Confounding variables adjusted	14 (64)	1 (100)	14 (74)	1 (20)

### 3.3. Prognostic risk factors

All prognostic factors are presented in Table 4. In total, 80 prognostic factors were found from the studies.

**Table 4:** Prognostic factors.

Category	Prognostic factor	Categorical (1) or continuous variable (2)	Evaluated in the study as [ref. number]			Predictive value in overall	Study quality (n)		
			Risk factor	Protective factor	Not significant statistically		Good	Fair	Poor
Personal factors and medical history	Age	1, 2	32,55	6	56,89,110,122,140	IE		7	1
	Female sex	1	110		32,55,89,122,140	Risk		5	1
	Body weight	1, 2	59,63,97		32,56,89,122	Risk		7	
	Body height	1	60			Risk		1	
	Body measures	1	59			Risk		1	
	Diabetes	1	57			Risk		1	
	Rheumatological event ≥1	1	32			Risk		1	
	Blood pressure	1		46,62		Protective	1	1	
	Pulse pressure	1		62		Protective		1	
	High cholesterol	1			61	NS		1	
	High HDL cholesterol	1			61	NS		1	
	High triglycerides	1			61	NS		1	
	Smoking and nicotine dependence	1	6,119		32,56	Risk		4	
	Alcohol dependence	1			119	NS		1	
	Psychoactive substance dependence	1			119	NS		1	
	Previous back surgery	1			18	NS		1	
	Previous episode of LBP	1	19,122			Risk		1	1
	Low back injured in MVC	1	99			Risk		1	
	Baseline disability before LBP	2	18		122	Risk		2	
	Baseline general health poor	2	18			Risk		1	
	Physical well-being	1		140	89	Protective		2	
	Physical exercise	1		97	32,56,89,110,122	Protective		5	1
	Level of education	1			88,110	NS			2
	Former productivity-related income	1	32			Risk		1	
	Disability compensation	1	55		18,19	Risk		2	1
	Occupational status	1			19,32,140	NS		2	1
	Number of different jobs held	1		32		Protective		1	
	Back pain in parents	1			110	NS			1
Symptom characteristics									
	Pain intensity	1, 2	54,55,122,140		89,110	Risk		4	1
	Pain duration	1	55		89,110,140	Risk		3	1
	Pain radiation	1			89,140	NS		2	
	Leg pain				55,88	NS		1	1
	To upper back		88			Risk			1
	Multiple pain sites				55	NS		1	
	Pain requiring medication	1			55,110,140	NS		2	1
	Days of reduced activity because of LBP	1		55		Protective		1	
	Affective pain	1			89	NS		1	
	Pain interfering sleeping	1			88	NS			1
	Pain worse on standing	1	19			Risk			1
	Pain worse on lying	1			19	NS			1
	Disability and functional limitation	1, 2	140		19,54,55,88,89,110	Risk		4	3
Biomechanical factors									
	Spinal mechanical load	2			6	NS		1	
	Work-related back pain	1	110			Risk			1
	Particularly physical work	1	56,58		110	Risk		2	1
	Physical intensity of work	1							
	Moderate or vigorous		83			Risk			1
	Vigorous only		83			Risk			1
	Frequent rest breaks from work	1			122	NS		1	
	Difficult working positions	1	56,83,103		32	Risk		2	2
	Repetitive short movements	1			103	NS			1
	Carrying heavy loads/lifting at work	1	32,58,83		56,103	Risk		3	2
	Working arms elevated	1			103	NS			1
	Bending and twisting trunk	1			103	NS			1
	Working kneeled/squatted	1			103	NS			1
	Vibration and jolts at work	1	56		103	Risk		1	1
	Working with animals	1			83	NS			1
	Working tired	1			83	NS			1
Psychological and psychosocial factors									
	Good quality of life	1		140		Protective		1	
	Mental well-being	1			89	NS		1	
	Depression	1, 2	55,119		32,89,110,140	Risk		5	1
	General anxiety	1	110,119		55	Risk		2	1
	Post-traumatic stress disorder	1	119			Risk		1	
	Antisocial personality disorder	1			119	NS		1	
	Any psychiatric diagnosis	1	119			Risk		1	
	Somatization	1	88,89			Risk		1	1
	Fear avoidance	1							
	In general				54	NS		1	
	Of work activity				89,110	NS		1	1
	Of physical activity				89,110	NS		1	1
	Perceived risk of persistence	1	55,88			Risk		1	1
	Catastrophizing	1	88		89	Risk		1	1
	Perceived stress	1	88			Risk			1
	Low tolerance of pain	1	88		55	Risk		1	1
	Coping by ignoring pain	1	88			Risk			1
	Coping by music or TV watching	1		88		Protective			1
	Nonrecognition of work	1	32			Risk		1	
	Job satisfaction/control	1			89	NS		1	
	Work absenteeism	1			89	NS		1	
	Support at work	1		88,89	32	Protective		2	1
	Support at home				89	NS		1	
	High psychological job demands	1			32,56,89,122	NS		4	
	Difficulty communicating	1			32	NS		1	

Categorical variable measured yes/no or in larger categories, continuous variable measured by continuous scale. Reference number of the studies evaluating each prognostic factor presented in brackets. The number of studies (sum) presented in quality categories.

HDL, high-density lipoprotein; IE, inconclusive evidence; LBP, low back pain; MCV, motor vehicle collision; NS, not significant statistically; protective, statistically significant protective factor; risk, statistically significant risk factor.

### 3.4. Personal factors and medical history

Three fair-quality studies found higher body weight to increase the risk of CLBP.^59,63,97^ Females seemed to be more at risk of developing chronicity according to 5 fair-quality studies^32,55,89,122,140^ and 1 poor-quality study,^110^ although statistical significance was achieved only in the latter. There was inconclusive evidence about age as a risk factor, although 2 fair-quality studies^32,55^ had a statistically significant result about age being a risk of chronicity. In 2 fair-quality studies, smoking and/or nicotine dependence was statistically significant risk factor.^6,119^ The only study rated as good quality found a statistically significant association between higher blood pressure and lower chronicity.^46^

### 3.5. Symptom characteristics

Higher pain intensity seemed to increase the risk of CLBP according to 6 studies,^54,55,89,110,122,140^ from which statistical significance was achieved in 4.^54,55,122,140^ Longer duration of symptoms before the onset of entering the studies (less than 3 months) was found to be predictive for chronicity in 1 fair-quality study.^55^ Seven studies investigated functional limitation and disability because of LBP as a risk factor,^19,54,55,88,89,110,140^ from which statistical significance was achieved in 1 study.^140^

### 3.6. Biomechanical factors

Carrying heavy loads at work was the most studied biomechanical risk factor for chronicity in 3 fair-quality studies^32,56,58^ and 2 poor-quality studies,^103,110^ and statistically significant in 3.^35,58,83^ Other significant factors predicting chronicity with statistical significance according to more than 1 study included particularly physical work^56,58^ and difficult working positions.^56,83,103^ Furthermore, vibrations and jolts at work significantly increased the risk of chronicity in 1 fair-quality study^56^ and nonsignificantly in 1 poor-quality study.^103^

### 3.7. Psychosocial factors

Numerous psychosocial factors were identified. Depression was the most studied factor predicting chronicity with statistically significant results in 2 studies^55,119^ and nonsignificantly in 4.^32,89,110,140^ Psychological risk factors that were investigated in more than 1 study included fear avoidance,^54,89,110^ general anxiety,^55,110,119^ somatization,^88,89^ pain catastrophizing,^88,89^ low tolerance of pain,^55,88^ patients' perceived risk of persistence of the symptoms,^55,88^ high psychological job demands,^32,56,89,122^ and finally support at work^32,88,89^ as a protective factor.

Compared with previous reviews,^15,133^ new factors were found to be predictive of CLBP. Of these, the most evident were obesity, smoking, higher pain intensity, and occupational factors, such as difficult working positions, vibrations, and jolts at work.

## 4. Discussion

The main findings in this review are that higher pain intensity, higher body weight, carrying heavy loads at work, difficult working positions, and depression are the most frequently observed prognostic risk factors for CLBP. Moreover, maladaptive behavior strategies, general anxiety, functional limitation during the episode, smoking, and particularly physical work are also explicitly predictive of chronicity. Most frequently observed protective factors were physical exercise and higher blood pressure.

According to the findings of this review, lifestyle-related factors, such as smoking and obesity, are major risk factors for pain chronicity. Odd ratios for smoking differed between 2.49 (95% confidence interval [CI] 1.15–5.40)^119^ and 4.41(95% CI 1.50–12.95).^6^ In obesity, odd ratios varied between 1.075 (95% CI 1.023–1.128)^59^ and 1.21 (95% CI 1.04–1.41)^97^ in women and between 1.091 (95% CI 1.027–1.158)^59^ and 1.16 (95% CI 1.05–1.29)^63^ in men. In general, the findings about the risk factors of pain chronicity are similar.^120,145^ Baseline personal factors concerning poorer general health^18^ and functionality^18^ were found to be significant risk factors for chronic pain in this review. Conversely, physical well-being^140^ and physical exercise^97^ were found to protect against chronicity. Poor general health and functionality are coherently interrelated to multimorbidity, which is a major risk factor for general pain chronicity.^24^ The same nonmodifiable risk factors, such as age and female sex, found in this review are also found to be risk factors for other chronic pain conditions.^28,41^

LBP-induced disability and functional limitation were significant risk factors according to the findings of this review.^140^ A study by Wand et al.^140^ reported that the correlation coefficient between Roland–Morris Disability Questionnaire and CLBP was 0.48. A similar finding about functional impairment at baseline was reported in a previous review.^15^ The lower levels of functionality might be a continuum of a person's lifestyle and behavioral factors. Therefore, avoiding bed rest despite the pain seems even more important.

The physical intensity of work, particularly strenuous physical work, carrying heavy loads, and working in difficult working positions, was related to higher chronicity in this review.^32,56,58,83,103^ In a study by Machado and colleagues,^83^ the carrying of heavy loads was predictive for CLBP with an odds ratio of 8.0 (95% CI 2.8–22.6). It is possible therefore that the physical work itself is preventing workers from getting back to work in a timely fashion^125^ and thereby contributing to the prolongation of the symptoms.

There is previous strong evidence that cognitive factors, such as attitudes, cognitive style, and fear-avoidance beliefs, are related to the development of pain and disability in patients with back pain.^82^ Maladaptive behaviors, such as perceived risk of persistence,^55,88^ pain catastrophizing,^88^ somatization,^88,89^ and coping by ignoring pain,^88^ were found to be risk factors in a total of 3 studies. It is not always the case that maladaptive behavior is the first step on the road to chronicity. The prospective designs included in this review would, however, implicate such causality, but one might suggest that fear avoidance, eg, is the immediate result of the pain in the acute phase of LBP, as Linton^82^ discussed in his review. Low tolerance of pain was a significant risk factor in this review.^88^ The low pain threshold is a complex concept and combines both genetic^124^ and psychological aspects. In a study of pain thresholds in patients with chronic pain, there was a correlation between lower pain threshold and depressive tendency and hypochondriac concerns.^75^

A previous history of LBP substantially increases the risk of a subsequent new episode.^105^ In this review, it was found to be a risk factor in 2 studies.^19,122^ Interestingly, we found no evidence of sleep disturbances being a risk factor for chronicity. However, since there is a bidirectional relationship between the intensity of LBP and sleep disturbances,^1^ one might assume it would also be a risk factor for CLBP. This would be an interesting hypothesis to study in the future.

So-called “yellow flags” is an umbrella term used to describe psychological risk factors and social and environmental risk factors for prolonged disability and failure to return to work as a consequence of musculoskeletal symptoms.^76^ Many of the risk factors for chronicity identified in this review fall under this category. The interest in yellow flags originates from the concept that early interventions might avert the development of disability. When patient selection is performed accurately and when an intervention known to address these factors is competently applied, good outcomes are to be expected.^96^

### 4.1. Limitations of this review

A major limitation of this review was that only 1 high-quality study was detected in our literature search. Loss to follow-up was significant in many fair-quality studies, and this reduced the number of good-quality studies. Furthermore, chronic low back pain as an outcome is hard to validate since it is always more or less self-reported. Many studies have tried to minimize this bias by using validated questionnaires.

Nine of the studies (36%) used the same population data from HUNT studies.^46,57–63,97^ The results that were only observed from HUNT studies were body height^60^ and measures,^59^ diabetes,^57^ blood pressure,^46,62^ and pulse pressure.^62^ However, the risk of bias in this particular study population can be assessed as low because of the large sample size and long follow-up period. The Nord-Trondelag Health Studies (HUNT studies) were population-based health surveys conducted in 1984 to 1986, 1995 to 1997, and 2006 to 2008. All residents older than 20 years of the entire Norwegian county were invited to take part in these large surveys.^63^

Some risk factors that seemed similar and were detected in multiple studies differed nonetheless to some extent in definition or measurement choice. To avoid too much heterogeneity inside 1 risk factor, they were intentionally not combined. Thus, it was difficult to reach a strong conclusion about the significance of several risk factors because they were only evaluated by a small number of studies.

Defining CLBP as persistent pain for at least 3 months is an artificial means of controlling the heterogenic population with LBP symptoms. Evidence from long-term studies indicates that people with long-term problems can have pain episodes separated by periods that are pain free, periods of continuous mild pain with low impact, or periods of severe pain with a large impact on their lives.^25^

When finding a potential association between a prognostic factor and an outcome, one must not assume that the effect is direct and isolated. Nonspecific low back pain is a multifactorial and complex condition with the impact of different factors changing over time.^32^ This review simply identifies the factors related to chronicity; it does not, however, study whether the presence of 1 factor is sufficient or whether a certain mix of factors is required. Therefore, when developing more comprehensive models that include connections between these factors, it is essential to consider which factors are truly important.

### 4.2. Usefulness of results and recommendations

A “wait and see” approach is no longer advisable because early screening provides reliable and valuable information for identifying those at risk of delayed recovery and for formulating a treatment strategy from the start.^81^ The subgrouping of patients with nonspecific LBP and finding tailored treatments and management strategies are the main research priorities in the field of LBP.^16^ It is therefore important to detect those patients at risk of developing chronicity in the early phases of the symptoms and to offer tailored treatment according to the risks in question. Especially stratification according to psychosocial risk factors has achieved promising results,^34,65^ but the disadvantage is the lack of work-related items, socioeconomic variables, and symptom factors. Then, additional steps may be needed to identify the specific problems of patients to improve outcomes.^81^

The findings of this review may be helpful in the planning of future studies concerning the prevention of CLBP and to aid clinicians detect patients at risk of chronicity.

## Disclosures

The authors have no conflicts of interest to declare.

## Appendix A. Supplemental digital content

Supplemental digital content associated with this article can be found online at http://links.lww.com/PR9/A99.
